# Exploring the Pharmacological Mechanism of Danzhi Xiaoyao Powder on ER-Positive Breast Cancer by a Network Pharmacology Approach

**DOI:** 10.1155/2018/5059743

**Published:** 2018-03-05

**Authors:** Kailin Yang, Liuting Zeng, Jinwen Ge

**Affiliations:** Hunan University of Chinese Medicine, Changsha, Hunan Province 410208, China

## Abstract

**Background:**

Breast cancer is the most common malignancy among women worldwide, but the long-term endocrine therapy is frequently associated with adverse side effects. Danzhi Xiaoyao powder (DXP) is a herbal formula that has an effect on breast cancer, especially ER-positive breast cancer. However, the active compounds, potential targets, and pharmacological and molecular mechanism of its action against cancer remain unclear.

**Methods:**

A network pharmacology approach comprising drug-likeness evaluation, oral bioavailability prediction, Caco-2 permeability prediction, multiple compound target prediction, multiple known target collection, breast cancer genes collection, and network analysis has been used in this study.

**Results:**

Four networks are set up—namely, ER-positive breast cancer network, compound-compound target network of DXP, DXP-ER-positive breast cancer network, and compound-known target-ER-positive breast cancer network. Some ER-positive breast cancer and DXP related targets, clusters, biological processes, and pathways, and several potential anticancer compounds are found.

**Conclusion:**

This network analysis successfully predicted, illuminated, and confirmed the molecular synergy of DXP for ER-positive breast cancer, got potential anticancer active compounds, and found the potential ER-positive breast cancer associated targets, cluster, biological processes, and pathways. This work also provides clues to the researcher who explores ethnopharmacological or/and herbal medicine's or even multidrugs' various synergies.

## 1. Introduction

Breast cancer is the most common malignancy among women all over the world whose mortality rate is the highest [1]. In China, breast cancer is also the most common one among Chinese women; the number of new breast cancer cases and the number of deaths in China account for 12.2% and 9.6% per year [2]. In the USA, the breast cancer cases account for approximately 32% (211,000 cases) of all new cancer cases per year, which has become the second leading cause of cancer death in US women [1]. The prognosis of newly diagnosed breast cancer patients is determined by the classification of breast cancer. There are at least four main subtypes of breast cancer according to different patterns of gene expression: luminal A, luminal B, the human epidermal growth factor receptor-2 (HER-2) overexpressing subtype, and the triple-negative subtype. Luminal A tends to have the best prognosis and luminal B includes estrogen receptor (ER) + and/or progesterone receptor (PGR) +, and HER2 + or HER2−, while the other 2 subtypes confer bad prognosis [3, 4]. ER-positive breast cancer includes luminal A subtype and luminal B subtype breast cancer. Research shows that approximately 70% of breast tumors are positive for ER expression at diagnosis [5]. The patients with ER-positive breast cancer are the suitable candidates of endocrine therapy. The endocrine therapy includes the selective estrogen receptor modulators (SERMs) such as tamoxifen (compete with estradiol for binding to the ER) and the selective estrogen receptor downregulators (SERDs) such as fulvestrant (inducing aromatase inhibitors [AI] and ER degradation), and it reduces the production of estrogen in peripheral tissues and tumors through inhibiting aromatase (an enzyme that synthesizes estrogen from testosterone and androstenedione). The tamoxifen is the pillar of endocrine therapy, whether in early or in advanced breast cancer patients; and, for the past three decades, it has been the most successful targeted therapy [6, 7].

However, the long-term endocrine therapeutic strategy is frequently associated with adverse side effects, which decrease patient compliance [8–10], such as the increased risk of endometrial cancer, thrombosis, and tumor metastasis [11–13]. Meanwhile, with the development of medicine, breast cancer treatment has entered a stage of diversified treatment. Therefore, adjuvant therapy, complementary and alternative medicine (CAM), becomes patients' selection. As a vital part of CAM, traditional Chinese medicine (TCM) may play an important role in it. The latest research shows that herbal medicine, a part of TCM, plays an important role in the whole breast cancer treatment; it can promote the recovery of postoperative patients or ones who receive radiotherapy or chemotherapy [14–18].

Danzhi Xiaoyao powder (DXP) is a herbal formula that comes from* Nei Ke Zhai Yao* wrote by Xue Jiand and it consists of eight herbs:* Cortex Moutan *(Dan Pi),* Gardeniae Fructus *(Zhi Zi),* Radix Bupleuri* (Chai Hu),* Angelicae Sinensis Radix* (Dang Gui),* Paeoniae Radix Alba* (Bai Shao),* Atractylodes Macrocephala Koidz.* (Bai Zhu),* Poria Cocos (Schw.) Wolf.* (Fu Ling), and* Licorice *(Gan Cao) [19]. In TCM theory, one of the etiologies of Ruyan (breast cancer) is the stagnation of Qi and phlegms cement in the breast and main and collateral channels astringent; and DXP is able to disperse stagnated liver Qi for relieving Qi stagnation. Thus, in clinical practice, DXP is mainly used for “liver depression transforming into fire” subtype Ruyan [20–22]. A number of basic researches also shows that DXP has a certain effect on breast cancer and precancerous lesions [19, 23], especially ER-positive breast cancer [23]. Therefore, DXP, alone or combined with other drugs (herbal formulae or western medicine), has the potential to be a drug for ER-positive breast cancer. However, its active compounds, the rationality of herbal combination, and pharmacological mechanism that inhibits the development, metastasis, and so on of breast cancer have not been clarified completely.

Herbal formulae can act on the multiple targets through their multiple components and play an integral role in the key biological process of disease development, which promotes the body back to equilibrium, and thus they play a therapeutic role [24]. However, many studies are still applying the traditional research idea, “one-drug-one-target-one-illness,” which ignores the multitarget and multicomponent characteristic of herbal formulae. Due to the rapid development of bioinformatics, the network pharmacology approach has become a new means to efficiently and systemically explore herbal formulae's or multiple drug combinations' molecular mechanism, evaluate their safety, and so on [25–27]. For instance, Tang et al. have used a network pharmacological methodology to explore the mechanism of certain herbal formula (XuanHuSuo Powder) for treating certain disease (osteoarthritis) [26]; and our previous research shows this methodology worked well in uncovering the mechanism of Xiaoyao Powder for anovulatory infertility [28] and in groping the mechanism of other herbal formulae for the other subtype of breast cancer [29]. Network pharmacology through systematic idea studies the relationships between drugs, targets, and diseases and shows the network of drug-targets by a visual way. Still, it abstracts the interaction relationship into a network model and studies the effect of drugs on a biological network from a holistic perspective [25, 30]. Therefore, the network pharmacology method is used for exploring the impact of DXP to ER-positive breast cancer and its pharmacological mechanism from another point of view.

## 2. Materials and Methods

### 2.1. Data Preparation

#### 2.1.1. Composite Compounds of DXP

To collect the compounds of DXP, we used the TCM Database@Taiwan [31] (http://tcm.cmu.edu.tw/zh-tw/), which is the most comprehensive TCM database in the world, and The Traditional Chinese Medicine Systems Pharmacology Database [32] (TCMSP, http://lsp.nwu.edu.cn/tcmsp.php), a unique system pharmacology platform designed for Chinese herbal medicines.

#### 2.1.2. Pharmacokinetic Prediction

Due to the disadvantages of biological experiments as being time-consuming and of high cost, identification of ADME (absorption, distribution, metabolism, and excretion) properties by in silico tools has now become an inevitable paradigm in pharmaceutical research. In this study, three ADME-related models, namely, the evaluation of oral bioavailability (OB), Caco-2 permeability, and drug-likeness (DL), are employed to identify the potential bioactive compounds of DXP [33].

*Oral Bioavailability*. OB prescreening is used to determine the fraction of the oral dose of bioactive compound which reaches systemic circulation in the TCM remedy. Here, a reliable in silico model OBioavail 1.1 [34] which integrates the metabolism (P450 3A4) and transport (P-glycoprotein) information was employed to calculate the OB values of herbal ingredients.

*Caco-2 Permeability*. The Caco-2 cell monolayers are widely applied as standard permeability-screening assay for prediction of the compound's intestinal absorption and fraction of the oral dose absorbed in humans [35]. The Caco-2 cell permeation values of all molecules are calculated by in silico model using the VolSurf approach [36].

*Drug-Likeness Evaluation*. Drug-likeness is a qualitative profile used in drug design to evaluate whether a compound is chemically suitable for the drug, and how drug-like a molecule is with respect to parameters affecting its pharmacodynamic and pharmacokinetic profiles which ultimately impact its ADME properties [37]. In order to identify drug-like compounds, we apply a database-dependent model using the Tanimoto coefficient to calculate the DL (see (1)) of each compound in DXP.

(1) $$\begin{array}{c} f\left(\;x,y\;\right)=\frac{xy}{{\left|x\right|}^{2}+{\left|y\right|}^{2}-xy}. \end{array}$$

*𝓍* represents the molecular parameters of herbal ingredients and *𝓎* represents the average molecular properties in DrugBank database (available online: http://www.drugbank.ca).

In this work, the compounds of OB ≥ 30%, Caco-2 > −0.4, and DL ≥ 0.18 are selected for subsequent research, and others are excluded.

According to these indexes, several compounds are included: ergosterol peroxide, ethyl oleate (NF), glabridin, glycyrrhetinic acid, linoleyl acetate, longikaurin A, mairin, mandenol, MOL000273, MOL001910, 508-02-1, 64997-52-0, 8*β*-ethoxy atractylenolide III, pachymic acid, paeonidanin, palbinone, saikosaponin C, beta-sitosterol, supraene, trametenolic acid, troxerutin, *α*-amyrin, MOL000285, 4-O-methylpaeoniflorin, glabrene, poricoic acid A, glycyrrhizin, sudan III, ZINC02816192, kaempferol, 7,9(11)-dehydropachymic acid, licochalcone G, paeoniflorgenone, areapillin, quercetin, stigmasterol, isoliquiritigenin, (+)-anomalin, isorhamnetin, vestitol, crocetin, 113269-36-6, *α*-spinasterol, licochalcone A, 113269-37-7, 3*β*-acetoxyatractylone, licoricone, 113269-39-9, petunidin, hederagenin, dehydroeburicoic acid, licochalcone B, ergosta-7,22E-dien-3beta-ol, MOL000280, MOL000287, mudanpioside H, NSC684433, octalupine, 18103-41-8, formononetin, 1-methoxyphaseollidin, paeoniflorin, glycyrin, ammidin, poricoic acid B, poricoic acid C, sainfuran, sitosterol, isoimperatorin, isolicoflavonol, cerevisterol, 3-methylkempferol, licoisoflavone B, cubebin, and (+)-catechin, 3′-hydroxy-4′-O-methylglabridin.

#### 2.1.3. Exceptional Molecules

In fact, reality constraints of most biological models force the dependent variables to lie in some finite bounds [33, 38], which may cause models to have some limitations, and our models are no exception. Thus, in order to avoid possible omissions of active ingredients during our prescreening process, we searched a large-scale text and selected oral absorbable compounds with pharmacological activity, in order to supplement the compounds.

According to the texts [39, 40], several compounds are found: ferulic acid, paeoniflorin sulfonate, albiflorin, senkyunolide I, and butylidenephthalide.

#### 2.1.4. Compound Target for DXP

Input all the active compounds into SciFinder (http://scifinder.cas.org), a database of chemical and bibliographic information attached to the Chemical Abstracts Service; and get the molecular structure of each active compound. Draw them in ChemBioDraw and save as “mol2” file format. Import these files into PharmMapper (http://lilab.ecust.edu.cn/pharmmapper/), which is a web server for potential drug-target identification using pharmacophore mapping approach [41].

#### 2.1.5. Known Targets

Acquire associated known target proteins with their target name, DrugBank ID, and validated status information from TCMSP. The targets proteins of TCMSP come from DrugBank and other databases and are almost the experimental validated drug-target pairs retrieved from HIT database and so on [32].

#### 2.1.6. Protein Name Correction

Because of the nonstandard naming, we use UniProtKB (http://www.uniprot.org/), which is the central hub for the collection of functional information on proteins, with accurate, consistent, and rich annotation. Input the protein names with the species limited to “Homo sapiens” and we can receive their official symbol. After these operations, proteins information of compound targets and known targets is obtained. The details are described in Tables S1 and S2. (See Supplementary Material.)

#### 2.1.7. ER-Positive Breast Cancer Targets

We collected different genes associated with ER-positive breast cancer from two resources. (1) Genecards (http://www.genecards.org): it is a database about genes, their products, and biomedical applications, which is maintained by Israel's Weizmann Institute of science. (2) OMIM database (http://omim.org/), which catalogues all known diseases with a genetic component and when possible links them to the relevant genes in the human genome and provides references for further research and tools for genomic analysis of a catalogued gene [42].

We searched these databases with keywords “luminal A breast cancer”, “luminal A type breast cancer”, “luminal B type breast cancer”, “luminal B breast cancer”, and “ER-positive breast cancer”, and got 85 genes totally. The details are described in Table S3.

#### 2.1.8. Protein-Protein Interaction Data

The data of protein-protein interaction (PPI) (such as the breast cancer target-compound target interaction) come from String [43] (http://string-db.org/, ver. 10), with the species limited to “Homo sapiens” and a confidence score >0.4, which is a database of known and forecasted protein-protein interactions.

### 2.2. Network Construction

#### 2.2.1. Network Construction Method

All the networks can be created via utilizing the network visualization software Cytoscape [44] (http://cytoscape.org/, ver. 3.4.0). It is the software that applies to visualizing biological pathways, intermolecular interaction networks, and many more. Furthermore, it supplies a basic set of features for data integration, analysis, and visualization for complicated network analysis.

Input the targets and the data of PPI into Cytoscape to construct different networks based on this research. Network construction was performed as follows: (1) ER-positive breast cancer network; (2) compound-compound target network of DXP; (3) DXP-ER-positive breast cancer network; (4) compound-known target-ER-positive breast cancer network.

#### 2.2.2. Cluster

The densely connected regions in large protein-protein interaction networks that may represent molecular complexes is defined as topological modules or clusters [45, 46], which has pure network property. Aggregation of nodes of similar or related function in the same network is called functional modules. A group of network components that together disrupt cellular function and then results in a particular disease phenotype are disease module. Due to the fact that the topology module, functional module, and disease module have the same meaning in the network, the functional module is equal to topology module and the disease can be regarded as the disturbance and destruction of functional model [45] The clusters of each network were obtained by analyzing the corresponding networks by MCODE, a plug-in of Cytoscape [46].

### 2.3. Enrichment Analysis

The Database for Annotation, Visualization and Integrated Discovery (DAVID, https://david-d.ncifcrf.gov, ver. 6.8) was applied for Gene Ontology (GO) enrichment analysis and pathway enrichment analysis [47]. The *P* value of each biological process and pathway is Modified Fisher Exact *P* Value, EASE score. The smaller, the more enriched. Therefore, the biological process/pathway with *P* < 0.05 were considered to be the significant biological process/pathway.

## 3. Results and Discussion

### 3.1. ER-Positive Breast Cancer Network Analysis

#### 3.1.1. ER-Positive Breast Cancer Network

**Figure 1:**
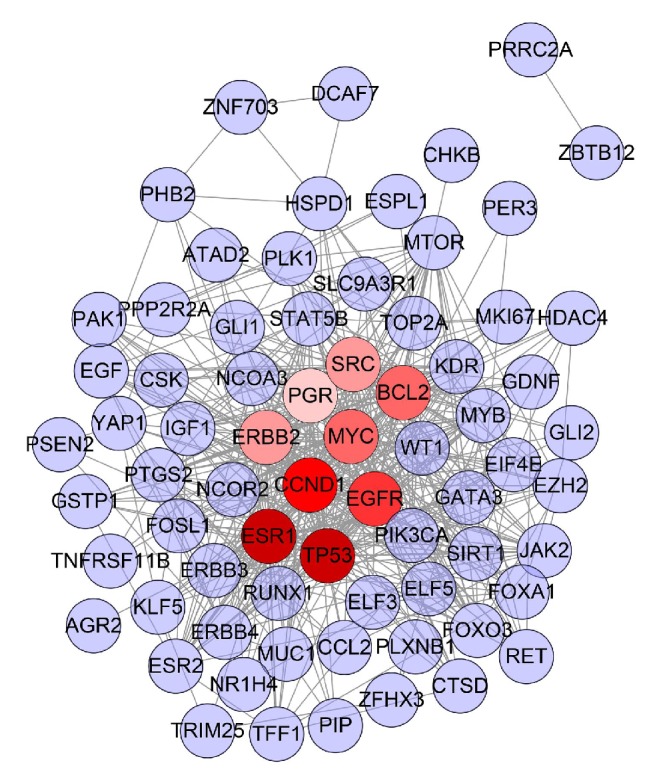
ER-positive breast cancer target PPI network.

Construct this “gene-gene interaction” network based on the data of ER-positive breast cancer genes' PPI and ER-positive breast cancer genes. This network contains 71 nodes and 497 edges (Figure 1).

In this network, the red nodes (ESR1, TP53, CCND1, EGFR, BCL2, MYC, SRC, ERBB2, and PGR) have higher degrees. The number of edges (which means the number of gene nodes that they are associated with) of each node is quite large (44 in ESR1 and TP53, 41 in CCND1, 40 in EGFR, 39 in BCL2 and MYC, 37 in SRC and ERBB2, 28 in PGR). This demonstrates that these genes are closely related to other genes in the network, suggesting that these genes may play an important role in ER-positive breast cancer. Pathogenic factors may directly influence ER-positive breast cancer-related genes or indirectly influence ER-positive breast cancer-related genes by affecting these genes, thereby affecting the development of ER-positive breast cancer, which suggests that these genes may be the key or central genes.

#### 3.1.2. Clusters of ER-Positive Breast Cancer Network

**Figure 2:**
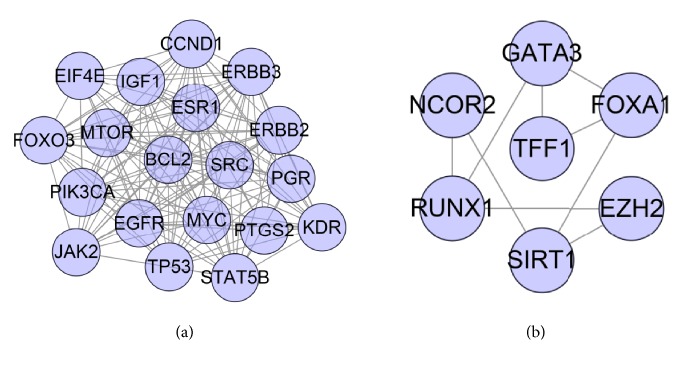
Cluster of ER-positive breast cancer target PPI network ((a), (b) stand for clusters 1 and 2).

Analyze the network by MCODE, and two clusters are returned (Table 1 and Figure 2). Input these clusters into DAVID for GO enrichment analysis, and several ER-positive breast cancer-related biological processes are returned. The details are described in Table S4. And, after filtering by *P* < 0.05, clusters 1 and 2 contained significant biological processes. The results are shown below.

Cluster 1 has (GO: 0018108) peptidyl-tyrosine phosphorylation, (GO: 0038128) ERBB2 signaling pathway, (GO: 0043066) negative regulation of apoptotic process, (GO: 0008283) cell proliferation, (GO: 0032355) response to estradiol, (GO: 0071364) cellular response to epidermal growth factor stimulus, (GO: 0048146) positive regulation of fibroblast proliferation, (GO: 0050679) positive regulation of epithelial cell proliferation, (GO: 0014068) positive regulation of phosphatidylinositol 3-kinase signaling, (GO: 0007169) transmembrane receptor protein tyrosine kinase signaling pathway, (GO: 0000165) MAPK cascade, (GO: 0038083) peptidyl-tyrosine autophosphorylation, (GO: 0007173) epidermal growth factor receptor signaling pathway, (GO: 0043406) positive regulation of MAP kinase activity, (GO: 0050727) regulation of inflammatory response, (GO: 0048010) vascular endothelial growth factor receptor signaling pathway, (GO: 0070374) positive regulation of ERK1 and ERK2 cascade, (GO: 0060397) JAK-STAT cascade involved in growth hormone signaling pathway, (GO: 0030330) DNA damage response, signal transduction by p53 class mediator, (GO: 0042523) positive regulation of tyrosine phosphorylation of Stat5 protein, (GO: 0030520) intracellular estrogen receptor signaling pathway, (GO: 0001525) angiogenesis, (GO: 0045737) positive regulation of cyclin-dependent protein serine/threonine kinase activity, (GO: 0034612) response to tumor necrosis factor, (GO: 0007259) JAK-STAT cascade, and (GO: 0000186) activation of MAPKK activity.

Cluster 2 gets (GO: 0032355) response to estradiol, (GO: 0071356) cellular response to tumor necrosis factor, and (GO: 0045766) positive regulation of angiogenesis.

In summary, we get the clusters through analyzing the network and get the biological processes of clusters through GO enrichment analysis; and, in analysis, two clusters and thirty-three biological processes were acquired. These biological processes are related to breast cancer; for example, some of them are related to limitless replicative potential like cell proliferation (GO: 0008283), positive regulation of ERK1 and ERK2 cascade (GO: 0070374), and tumor promotion inflammation, such as regulation of inflammatory response (GO: 0050727) and response to tumor necrosis factor (GO: 0034612), while others are associated with the response to stimulation of breast cancer, such as response to estrogen (GO: 0032355). Pathogenic factors may directly or indirectly affect the genes enriched to each biological process so as to influence these ER-positive breast cancer-related biological processes, which thereby affect the development of ER-positive breast cancer. In this network analysis, we can observe the molecular mechanism of the development of ER-positive breast cancer more clearly.

Recent research shows that ER (including ER*α* and ER*β*), as the member of the nuclear receptor superfamily of transcription factor, takes part in the development of breast cancer. One of the important roles of ER is to regulate the expression of intracellular oncogene and tumor suppressor genes; only approximately 10% of normal breasts express estrogen receptors, but 60% to 70% of breast cancer express estrogen receptors [48, 49]. The study shows that the ER*α*/ER*β* ratio in ER-positive breast cancer tissue is higher than that in surrounding normal breast tissue, which shows the expression of ER*α* increased and ER*β*'s decreased [50]. In breast cancer, nuclear estrogen receptor- (nER-) mediated genomic signaling pathway leads to breast-associated oncogene activation or tumor suppressor gene inactivation by modulating transcription of target genes. Its mediated modes include classic estrogen responsive element (ERE) mode (acting on the ERE in the target gene promoter region), non-ligand-dependent genome mode, and non-ERE-dependent genome mode.

In classic ERE mode, unbound ER can be nuclear localized and bound loosely to the ERE [51, 52]. However, binding of estradiol to the ER initiates a cascade of events leading to strong ER dimerisation, increased nuclear localization and binding to ERE in the regulatory regions of target genes, and gene transcription (mediated by AF1 and AF2 of the ER). AF1, located in domains A and B of the amino terminal of the ER, is activated by growth factors acting through the mitogen-activated protein kinase (MAPK) pathway, while AF2, located in domain E of the carboxyterminal of the ER ligand-binding domain, is activated by estradiol [53].

In non-ligand-dependent genome mode, in the absence of estrogen, the growth factor-activated intracellular signaling pathway induces nER to bind to ERE to regulate gene transcription; the epidermal growth factor (EGF) activates the MAPK signaling pathway through the EGF receptor and then phosphorylates the nER, which allows nER to be activated in the absence of estrogen [54]. Insulin-like growth factor (IGF) and tumor transforming growth factor-*α* (TGF-*α*) can also activate nER through inducing MAPK cascade [53, 55, 56]. Research shows that ERs being activated by estrogen can induce MCF-7 breast cancer cell intracellular cascade signal response, including IGFR activation, and then produces matrix metalloproteinases, which thereby promotes the release of heparin-binding EGF-like growth factor (HB-EGF) and activate the EGF receptor- (EGFR-) induced MAPK signaling pathway [53, 55, 56]. Bartucci et al. have reported that, in ER-positive breast cancer, IGF-1 receptor (IGF1R) and insulin receptor substrate 1 (IRS1) are also highly expressed, and their high expression is associated with radiotherapy tolerance and tumor recurrence [57]. Oesterreich et al. have reported that, in the MCF-7 cell, IGF-1 can induce the phosphorylation of MAPK and phosphoinositide 3-kinase (PI3K) [58]. Also, in this estrogen-driven human breast cancer cell, there is a synergistic effect between c-Myc and TGF-*α*; this synergistic effect involves TGF-*α*-induced Bcl-XL, and Bcl-XL can block c-Myc-induced apoptosis [59].

In nonclassical pathways of ERE signaling, ERs can stimulate gene transcription by interacting with other transcription factors bound to promoters of responsive genes. Genes that are regulated by estrogen but do not have an ERE include cyclin D1, p21, and the PR [60]. Many such genes are regulated by ER interactions with stimulating protein 1 (Sp1) and the signal transducers and activators of transcription (STAT) family of transcription factors and transcription factors associated with activating protein 1 (AP1) sites [61].

In breast cancer, membrane estrogen receptor- (mER-) mediated signaling can make estradiol exert early signaling events within target cells minutes after stimulation, which makes it likely that these effects are independent of transcription. The activation of mER not only leads to activation of MAPK signaling and PI3K signaling and a rise in cytoplasmic calcium levels but also activates the release of EGF and IGF, thus leading to stimulation of EGFR and IGFR, respectively [48]. In addition, G-protein coupled estrogen receptor (GPR30) can act independently of ER*α* and ER*β* and is linked to estrogen-mediated regulation of the EGFR to MAPK signaling axis by stimulation of adenylyl cyclase activity [56, 62].

The absence of ER is a sign of poor prognosis, and the status of coactivators and corepressors of ER in the nucleus also greatly affects the pathogenic ability of estrogen in breast cancer cells [54, 63, 64]. Furthermore, progesterone also plays an important role in breast cancer. The progesterone receptor (PR) status significantly improves prediction of outcome over ER status alone for adjuvant endocrine therapy [65]. In particular, compared with patients with ER+/PR+ tumor, tamoxifen-treated patients with ER+/PR− tumor show a significantly higher relative risk of recurrence and death. Latest studies demonstrate that activation of growth factor receptor (GFR) pathways can lead to decreased PR expression, which provides evidence for a role of GFR signaling in endocrine treatment response [66]. Actually, the bidirectional crosstalk between steroid receptors and receptor tyrosine kinases is a major determinant of endocrine resistance. Because of that the overexpression of EGFR family members (EGFR and HER2) and IGF1R has been associated with tamoxifen resistance [67].

#### 3.1.3. Pathway of ER-Positive Breast Cancer Network

**Figure 3:**
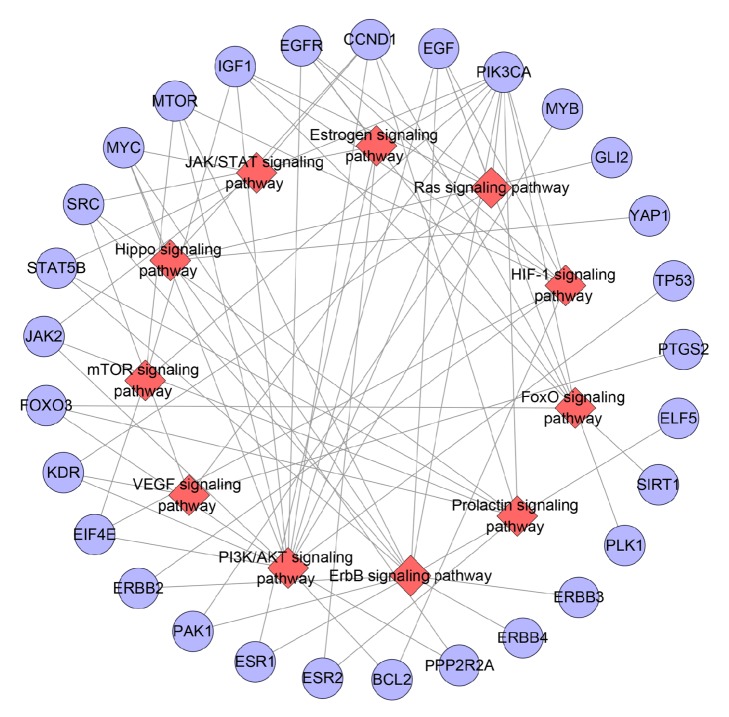
Pathway of ER-positive breast cancer target PPI network (blue circle stands for breast cancer gene; red diamond stands for pathway).

Input all of the genes into DAVID to do pathway enrichment analysis and get sixteen significant breast cancer associated pathways (Figure 3). PI3K/AKT signaling pathway contains 15 genes, which is the most; ErbB signaling pathway includes 11 genes; Prolactin signaling pathway has 9 genes; both HIF-1 signaling pathway and FoxO signaling pathway get 8 genes; and so on. The details are described in Table S5.

These pathways with so many breast cancer associated genes may be the key pathways in breast cancer's development. We can find that these pathways have several of the same genes (e.g., both estrogen signaling pathway and VEGF signaling pathway have SRC and PIK3CA). Meanwhile, the downstream effects of these signaling pathways are also complex and multiple, suggesting that different signal pathways are closely related and have great complexity. Intervening in these 16 signaling pathways may be the potential strategy of treating ER-positive breast cancer in the future.

As a systemic disease, the abnormality of the signaling of hormone, cytokines, growth factors, and so on may lead to excessive amplification of certain genes and thus causes normal cells to receive abnormal proliferation, differentiation, and growth signals, which ultimately promote normal cell carcinogenesis. Meanwhile, their respective mediated signaling pathways cross each other to jointly promote the occurrence, invasion, and metastasis of breast cancer. The study finds that the rapid nongenomic effect of estrogen is activating the PI3K/AKT pathway by stimulating IGF-1R in breast cancer cell so as to lead to increased mitosis of breast cancer cells. In breast cancer cells, the utilization of ICI182780 (Faslodex), an estrogen receptor antagonist, can reduce the response of EGF, suggesting that, among ER, EGFR, and IGFR signaling pathways, there is not a single linear relationship, but the interaction with each other [68].

In the cell, there are multiple signaling pathways associated with the development, invasion, and metastasis of breast cancer, such as receptor tyrosine kinase- (RTK-) mediated signaling pathways, estrogen-regulated ER signaling pathway, TGF-*β* signaling pathway, Wnt signaling pathway, and so on. Besides these pathways, there are also other pathways involved in breast cancer stem cell self-renewal, proliferation, and differentiation, such as Hippo signaling pathway; the Wnt signaling pathway is also associated with the self-renewal, proliferation, and differentiation of breast cancer stem cell [69].

The estrogen-regulated signaling pathways mainly contain the PI3K/AKT signaling pathway, MAPK/ERK signaling pathway, cAMP/PKA signaling pathway, and JNK signaling pathway. These pathways are thought to have the role of regulating the growth, development, and cell proliferation of mammary glands. Meanwhile, they are also the molecular basis of normal mammary gland tissue transformation, uncontrolled proliferation, and antiapoptosis. TGF-*β* signaling pathway can promote tumor cell metastasis by affecting tumor microenvironment and enhancing invasiveness and inhibiting the function of immune cells. The RTK-mediated signaling pathways are mainly ERK/MAPK signaling pathway, PI3K/PKC/IKK signaling pathway, and the PI3K/PTEN/AKT pathway. These pathways are necessary for the proliferation and differentiation of mammary epithelial cells, and they also determine the poor differentiation and heterogeneity of breast cancer cells.

As a highly heterogeneous disease, multiple antiapoptotic pathways associated with inflammatory pathways and certain signaling molecules also mediate the development of breast cancer. For instance, MAPK/ERK1/2 pathway, NF-*κ*B, and so on play a novel role in the breast cancer cell response to a metabolic stress [70].

### 3.2. Compound-Compound Target Network Analysis

**Figure 4:**
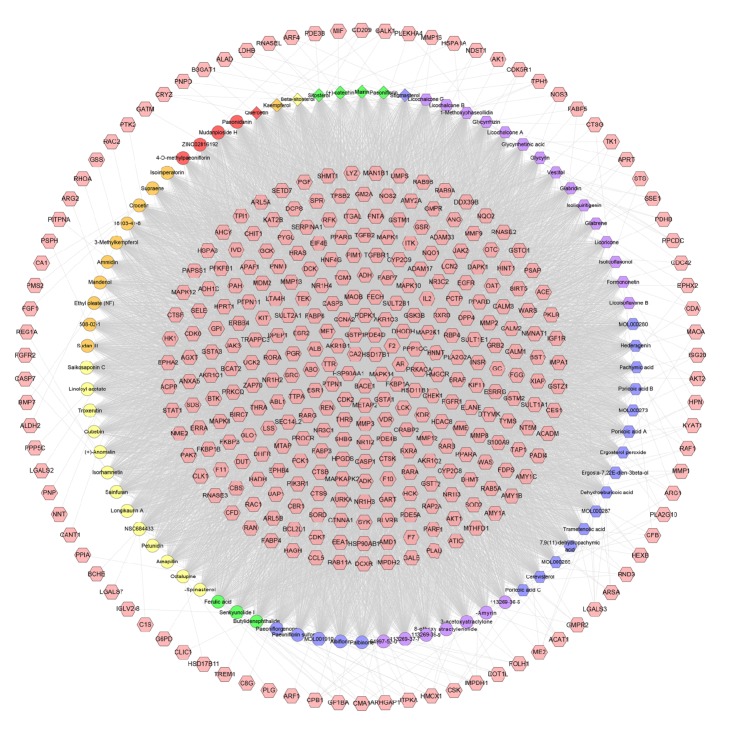
Compound-compound target network of DXP consists of 374 compound targets and 87 compounds (pink hexagon stands for compound targets; red, orange, yellow, green, blue, and purple circles stand for compounds of* Cortex Moutan, Gardeniae Fructus*,* Radix Bupleuri*,* Angelicae Sinensis Radix*,* Paeoniae Radix Alba*, and* Atractylodes Macrocephala Koidz.*, respectively. Blue and purple hexagons stand for compounds of* Poria Cocos (Schw.) Wolf.* and* Licorice*. Blue diamond stands for common compound of* Gardeniae Fructus*,* Radix Bupleuri,* and* Angelicae Sinensis Radix. *Green diamonds stand for compounds of* Cortex Moutan* and* Paeoniae Radix Alba*. Yellow diamond stands for common compound of* Gardeniae Fructus*,* Angelicae Sinensis Radix,* and* Paeoniae Radix Alba*. Orange diamond stands for common compound of* Cortex Moutan*,* Gardeniae Fructus*,* Radix Bupleuri,* and* Paeoniae Radix Alba*. Red diamond stands for common compound of* Cortex Moutan*,* Gardeniae Fructus,* and* Radix Bupleuri*).

This network is composed of 454 nodes (374 compound target nodes and 80 compound nodes) and 10371 edges. In this network we can find that many targets are hit by multiple compounds (central nodes, e.g., HSD17B1, CA2, MAPK14, BACE1, and HSP90AA1, can be hit by all compounds), but some can be modulated by only one compound (peripheral nodes, e.g., FGFR2, ALDH2, PPIA, and IGLV2-8). In other words, these compounds are able to regulate the compound targets. For example, all (+)-catechin, ammidin, dehydroeburicoic acid, octalupine, and paeoniflorin can regulate ESR1, ESR2, PGR, and IGF1R (Figure 4).

This suggests that DXP's compounds may act on these targets and thus play a pharmacological role in other diseases besides breast cancer, which invisibly shows herbal formulae's feature of multicompound-multitarget-multidisease. Its potential effect can be carried out by this network. However, we do not know whether the relationship between them is synergistic, antagonistic, or otherwise. Therefore, it needs further research.

### 3.3. DXP-ER-Positive Breast Cancer Network Analysis

#### 3.3.1. DXP-ER-Positive Breast Cancer Network

**Figure 5:**
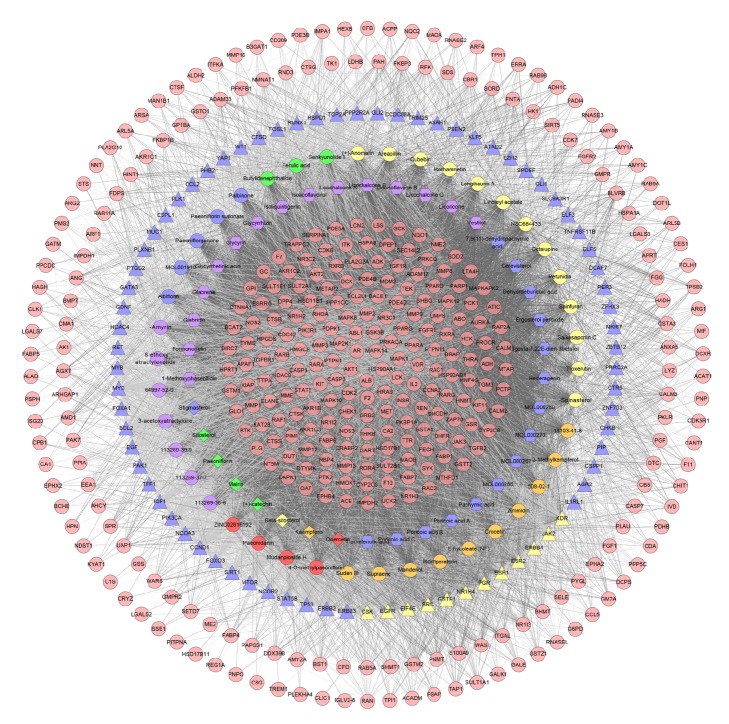
DXP-ER-positive breast cancer network (the representation of red, orange, yellow, green, blue, and purple circle, purple and blue hexagon, and blue, green, yellow, orange, and red diamonds is the same as Figure 4. Pink circle, blue triangle, and yellow triangles stand for compound target, breast cancer target, and compound-ER-positive breast cancer target. Dark lines stand for the relationship between compounds and other nodes, and light lines stand for relationship between breast cancer targets, compound-ER-positive breast cancer target, and compound targets).

Integrating ER-positive breast cancer network and compound-compound target network, we can get DXP-ER-positive breast cancer network. This network contains 519 nodes and 15855 edges. Compared with ER-positive breast cancer network, this network adds 448 nodes and 15385 edges (Figure 5).

#### 3.3.2. Clusters of DXP-ER-Positive Breast Cancer Network

**Figure 6:**
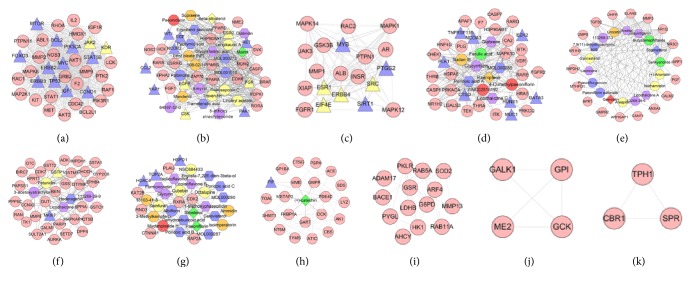
Cluster of DXP-ER-positive breast cancer network ((a), (b), (c), (d), and so on stand for clusters 1, 2, 3, 4, and so on. The representation of red, orange, yellow, green, blue, and purple circle, purple and blue hexagon, and blue, green, yellow, orange, and red diamonds is the same as Figure 4. Pink circle, blue triangle, and yellow triangles stand for compound target, breast cancer target, and compound-ER-positive breast cancer target. Dark lines stand for the relationship between compounds and other nodes, and light lines stand for relationship among breast cancer targets, compound-ER-positive breast cancer target and compound targets).

Analyze the network by MCODE, and eleven clusters are returned. Cluster 2 gets ergosterol peroxide, ethyl oleate (NF), glabridin, glycyrrhetinic acid, linoleyl acetate, longikaurin A, mairin, mandenol, MOL000273, MOL001910, 508-02-1, 64997-52-0, 8*β*-ethoxy atractylenolide III, pachymic acid, paeonidanin, palbinone, saikosaponin C, beta-sitosterol, supraene, trametenolic acid, troxerutin, and *α*-amyrin. Cluster 4 has ferulic acid, MOL000285, 4-O-methylpaeoniflorin, glabrene, poricoic acid A, glycyrrhizin, sudan III, ZINC02816192, kaempferol, and licochalcone G. Cluster 5 gets 7,9(11)-dehydropachymic acid, paeoniflorgenone, paeoniflorin sulfonate, areapillin, quercetin, senkyunolide I, stigmasterol, butylidenephthalide, isoliquiritigenin, (+)-anomalin, isorhamnetin, vestitol, crocetin, 113269-36-6, *α*-spinasterol, licochalcone A, 113269-37-7, and licoricone. Cluster 6 includes 3*β*-acetoxyatractylone, 113269-39-9, petunidin, hederagenin, and licochalcone B. Cluster 7 has dehydroeburicoic acid, ergosta-7,22E-dien-3beta-ol, MOL000280, MOL000287, mudanpioside H, NSC684433, octalupine, 18103-41-8, formononetin, albiflorin, 1-methoxyphaseollidin, paeoniflorin, glycyrin, ammidin, poricoic acid B, poricoic acid C, sainfuran, sitosterol, isoimperatorin, isolicoflavonol, cerevisterol, 3-methylkempferol, licoisoflavone B, and cubebin. Cluster 8 includes (+)-catechin. These clusters get a total of 80 compounds that appears in the compound-compound target network, which suggests that these compounds, even the whole herbal formula, Danzhi Xiaoyao powder, play important roles in treating ER-positive breast cancer (Table 2 and Figure 6).

In addition, there are some breast cancer-related genes in clusters. Cluster 1 gets MTOR, BCL2, FOXO3, ERBB3, ERBB2, TP53, MYC, PI3KCA, IGF1, CCND1, STAT5B, JAK2, and KDR. Cluster 2 has ESR2, CSK, PGR, EGFR, CCL2, WT1, YAP1, NCOR2, PAK1, EGF, and RET. Cluster 3 includes EIF4E, SRC, ERBB4, ESR1, SIRT1, MYB, and PTGS2. Cluster 4 gets PLK1, CTSD, RUNX1, EZH2, TNFRSF11B, NCOA3, GATA3, and MUC1. Cluster 6 has GSTP1 and MKI67. Cluster 7 gets HSPD1, TOP2A, and HDAC4. Cluster 8 includes PIP. These genes may be the key genes of DXP treating ER-positive breast cancer.

Deal with these clusters by GO enrichment analysis. Clusters 8, 10, and 11 do not return ER2-positive breast cancer-related biological processes. And, after filtering by *P* < 0.05, only clusters 1, 2, 3, and 4 contained significant biological processes. Take clusters 1, 2, and 4 as examples, and the results are shown below.

Cluster 1 gets (GO: 0048015) phosphatidylinositol-mediated signaling, (GO: 0014066) regulation of phosphatidylinositol 3-kinase signaling, (GO: 0018108) peptidyl-tyrosine phosphorylation, (GO: 0000165) MAPK cascade, (GO: 0014068) positive regulation of phosphatidylinositol 3-kinase signaling, (GO: 0048010) vascular endothelial growth factor receptor signaling pathway, (GO: 0038128) ERBB2 signaling pathway, (GO: 0038083) peptidyl-tyrosine autophosphorylation, (GO: 0007169) transmembrane receptor protein tyrosine kinase signaling pathway, (GO: 0008283) cell proliferation, (GO: 0007173) epidermal growth factor receptor signaling pathway, (GO: 0048009) insulin-like growth factor receptor signaling pathway, (GO: 0042523) positive regulation of tyrosine phosphorylation of Stat5 protein, (GO: 0050731) positive regulation of peptidyl-tyrosine phosphorylation, (GO: 0043552) positive regulation of phosphatidylinositol 3-kinase activity, (GO: 0071364) cellular response to epidermal growth factor stimulus, (GO: 0001525) angiogenesis, (GO: 0036092) phosphatidylinositol-3-phosphate biosynthetic process, (GO: 0007259) JAK-STAT cascade, (GO: 0070374) positive regulation of ERK1 and ERK2 cascade, (GO: 0007265) Ras protein signal transduction, (GO: 0060070) canonical Wnt signaling pathway, (GO: 0032355) response to estradiol, (GO: 0033209) tumor necrosis factor-mediated signaling pathway, (GO: 0060644) mammary gland epithelial cell differentiation, (GO: 0008631) intrinsic apoptotic signaling pathway in response to oxidative stress, (GO: 0030330) DNA damage response, signal transduction by p53 class mediator, (GO: 0060397) JAK-STAT cascade involved in growth hormone signaling pathway, and (GO: 0043123) positive regulation of I-kappaB kinase/NF-kappaB signaling.

Cluster 2 has (GO: 0043401) steroid hormone mediated signaling pathway, (GO: 0001525) angiogenesis, (GO: 0006954) inflammatory response, and so forth.

Cluster 4 includes (GO: 0043401) steroid hormone mediated signaling pathway, (GO: 0043627) response to estrogen, (GO: 0008285) negative regulation of cell proliferation, (GO: 0043410) positive regulation of MAPK cascade, (GO: 0070374) positive regulation of ERK1 and ERK2 cascade, and (GO: 0000165) MAPK cascade.

Analyzing another cluster by the same way, we get the same related biological processes. The details are described in Table S6.

Overall, we obtain the biological processes through analyzing the network and GO enrichment analysis clusters; and, in analysis, eleven clusters and forty-five biological processes were acquired. These biological processes are associated with breast cancer; for instance, some of them are associated with sustained angiogenesis (e.g., GO: 0048010 and GO: 0001525), while others are related to the signal response, such as ERBB2 signaling pathway (GO: 0038128) and response to estradiol (GO: 0032355); there are also some biological processes related to limitless replicative potential, like cell proliferation (GO: 0008283) and regulation of phosphatidylinositol 3-kinase signaling (GO: 0014066). Beside these, there are many other biological processes associated with breast cancer characteristics; and all of them can be found in Table S6, and their details are described in it, too. The compounds of DXP in each cluster may regulate the protein or gene nodes enriched to biological processes directly or indirectly so as to affect the ER-positive breast cancer associated biological processes. This thereby influences the development of ER-positive breast cancer indirectly so as to achieve DXP's therapeutic effect.

#### 3.3.3. Pathway of DXP-ER-Positive Breast Cancer Network

**Figure 7:**
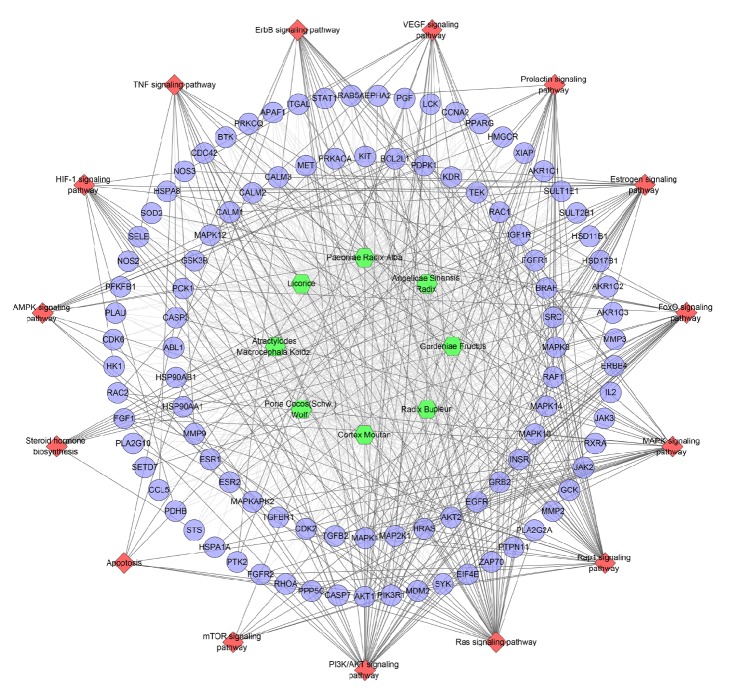
Pathway o of DXP-ER-positive breast cancer network (blue circle stands for compound target; red diamond stands for pathway; green hexagon stands for herb).

Importing all targets into DAVID, we can get fifteen ER-positive breast cancer-related significant pathways (Figure 7). In Figure 7,* Cortex Moutan* hits the most genes (90 genes), which indicates that this herb may be the main herb in treating ER-positive breast cancer. In other words, in TCM theory, people always call it the monarch herb (Jun herb) in DXP when it was used for treating ER-positive breast cancer. In addition,* Radix Bupleuri* regulates 87 genes and it is in the second place, which indicates that this herb may assist* Cortex Moutan *to play the role. And, in TCM theory, people may call it the minister herb (Chen herb). Meanwhile, PI3K/AKT signaling pathway has the most genes (38 genes); Ras signaling pathway has 37 genes; Rap1 signaling pathway has 31 genes; MAPK signaling pathway has 27 genes; FoxO signaling pathway has 24 genes; and so on. These pathways may be the key pathway to DXP treating ER-positive breast cancer. The details are described in Table S7.

DXP, as a multiherb formula, can act on multiple ER-positive breast cancer-related targets by its multiple components in each herb. For example, ammidin, dehydroeburicoic acid, (+)-catechin, paeoniflorin, and so on can regulate ESR1, ESR2, and PGR; this can be observed in Figures 4 and 5 and so on; and we need further research to study whether the relationship between them is synergistic, antagonistic, or otherwise. Through the MCODE, several clusters are returned and some of the clusters get the compounds coming from each herb in DXP. With the deepening of research, some of these components of the pharmacological effects have been reported. The following is an example.

For the compounds in cluster 2, ergosterol peroxide is able to kill MCF7 by inducing apoptosis [71]. Glabridin can inhibit migration, invasion, and angiogenesis of MDA-MB-231 human breast adenocarcinoma cells by inhibiting focal adhesion kinase/Rho signaling pathway [72]. Pachymic acid can suppress nuclear factor-*κ*B-dependent matrix metalloproteinase-9 expression to impair breast cancer cell invasion [73]. Beta-sitosterol can activate Fas signaling in human breast cancer cells; it can also enhance tamoxifen effectiveness on breast cancer cells by affecting ceramide metabolism [74].

For the compounds in cluster 4, kaempferol is a phytoestrogen that can suppress triclosan-induced epithelial-mesenchymal transition and metastatic-related behaviors of MCF-7 breast cancer cells [75].

For the compounds in cluster 5, quercetin can influence the Akt/AMPK/mTOR signaling; thus it has the potential of being anti-breast cancer [76]; also, gold nanoparticle-conjugated quercetin is able to affect the EGFR/VEGFR2-mediated pathway so as to inhibit epithelial-mesenchymal transition, angiogenesis, and invasion [77]. Isoliquiritigenin can prevent mammary carcinogenesis by inhibiting breast cancer stem cells through WIF1 demethylation [78]. Isorhamnetin can inhibit cell proliferation and induces apoptosis in breast cancer for it influences AKT and MEK signaling pathways [79]. Crocetin is able to downregulate matrix metalloproteinases to inhibit invasion of MDA-MB-231 breast cancer cells [80].

For the compounds in cluster 6, licochalcone B can upregulate the expressions of Caspase 3, Caspase 9, Bax, Cyclin A, Cdk2, Cdc25 A, and so on and downregulate the expression of Bcl-2, p21, and so on to arrest cell cycle progression and induces apoptosis in MCF-7 cells [81]. Formononetin is a naturally existing isoflavone and it can inhibit migration and invasion of MDA-MB-231 breast cancer cells through PI3K/AKT signaling pathways [82].

However, there are still lots of compounds' pharmacological effects in DXP that need to be clarified. This analysis may provide some clue, such as important compounds and central targets (genes and proteins), for researchers who want to grope the pharmacological or molecular mechanism of the compounds in whole formula or in each herb.

### 3.4. Compound-Known Target-ER-Positive Breast Cancer Network Analysis

#### 3.4.1. Compound-Known Target Network

This network consists of 280 nodes (225 compound target nodes and 55 compound nodes) and 877 edges. The compound-known target network is smaller than the compound-target network. These known targets come from DrugBank and so on and have been reported in the literature. These networks are set up for confirming and supplementing DXP's effect on ER-positive breast cancer.

#### 3.4.2. Compound-Known Target-ER-Positive Breast Cancer Network

**Figure 9:**
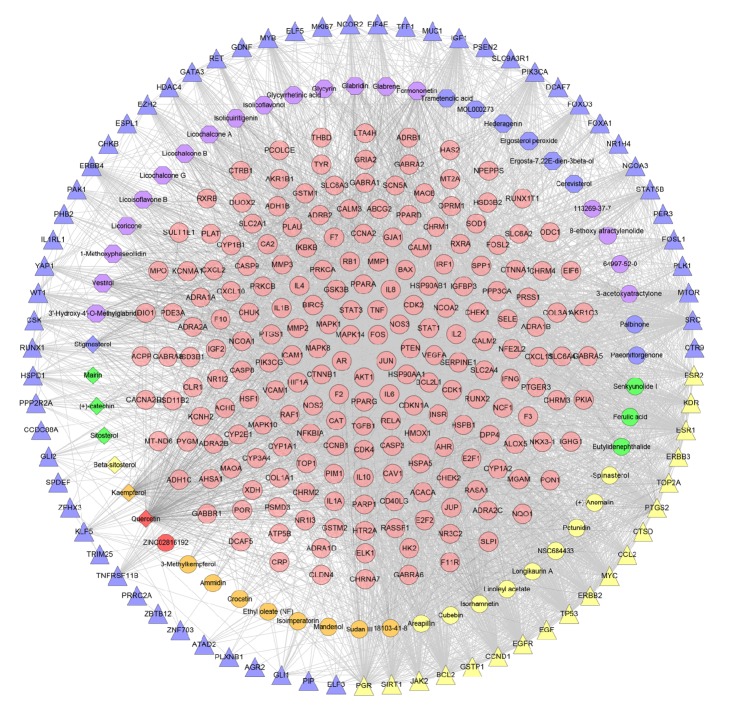
Compound-known target-ER-positive breast cancer network (blue hexagon stands for known target. The representation of red, orange, yellow, green, blue, and purple circle, blue and purple octagon, and blue, green, yellow, orange, and red diamonds is the same as Figure 8. Pink circle, blue triangle, and yellow triangle stand for known target, breast cancer target, and known-ER-positive breast cancer target. Dark lines stand for relationship between compounds and known targets, and light lines stand for relationship between breast cancer targets, known-ER-positive breast cancer target, and know targets).

Compound-known target-ER-positive breast cancer network is composed of 336 nodes and 5536 edges (Figure 9). This network is a confirmation network of DXP-ER-positive breast cancer network. In these known targets which are validated by experiments, we find some compound targets (predicted targets) and compound-ER-positive breast cancer target, which not only prove the reliability of predictive target but also suggest the effectiveness of DXP on the disease such as breast cancer. There are also some known targets that do not coincide with compound target; according to this, we assume that these targets may be regulated by compound target indirectly.

#### 3.4.3. Cluster and Pathway of Compound-Known Target-ER-Positive Breast Cancer Network

**Figure 10:**
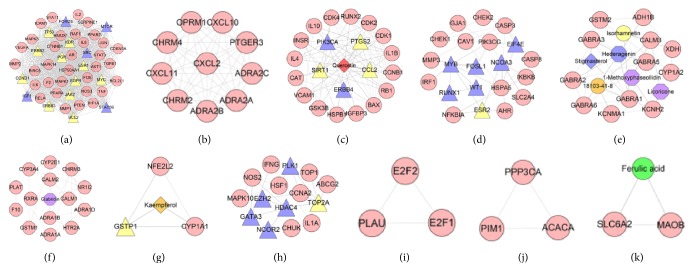
Cluster of compound-known target-ER-positive breast cancer network ((a), (b), (c), (d), and so on stand for clusters 1, 2, 3, 4, and so on. The representation of red, orange, yellow, green, blue, and purple circle, blue and purple octagon, and blue, green, yellow, orange, and red diamonds is the same as Figure 8. Pink circle, blue triangle, and yellow triangle stand for known target, breast cancer target, and known-ER-positive breast cancer target. Dark lines stand for relationship between compounds and known targets, light lines stand for relationship between breast cancer targets, known-ER-positive breast cancer target, and known targets).

Analyzing the network by MCODE, eleven clusters are returned (Table 3 and Figure 10).

In this analysis, we get several compounds (quercetin, hederagenin, stigmasterol, 1-methoxyphaseollidin, licoricone, isorhamnetin, 18103-41-8, glabridin, kaempferol, and ferulic acid) and several ER-positive breast cancer genes (KDR, BCL2, ESR1, ERBB3, JAK2, MYC, ERBB2, TP53, EGFR, PGR, CCND1, IGF1, FOXO3, STAT5B, MTOR, SRC, PTGS2, SIRT1, CCL2, PIK3CA, ERBB4, ESR2, EIF4E, NCOA3, FOSL1, RUNX1, WT1, MYB, GSTP1, TOP2A, NCOR2, PLK1, EZH2, HDAC4, and GATA3). Most of them are the same as those in the clusters of DXP-ER-positive breast cancer network, which indirectly confirm DXP's effects.

Deal with these clusters by GO enrichment analysis. Clusters 5, 7, 10, and 11 do not return ER-positive breast cancer-related biological processes. And, after filtering by *P* < 0.05, only clusters 1, 2, 3, 4, 6, 8, and 9 contained significant biological processes. Take clusters 1 and 6 as examples, and the results are shown below.

Cluster 1 has (GO: 0043066) negative regulation of apoptotic process, (GO: 0008284) positive regulation of cell proliferation, (GO: 0071456) cellular response to hypoxia, (GO: 0050731) positive regulation of peptidyl-tyrosine phosphorylation, (GO: 0008283) cell proliferation, (GO: 0070374) positive regulation of ERK1 and ERK2 cascade, (GO: 0032355) response to estradiol, (GO: 0008285) negative regulation of cell proliferation, (GO: 0001525) angiogenesis, (GO: 0050679) positive regulation of epithelial cell proliferation, (GO: 0000165) MAPK cascade, (GO: 0038128) ERBB2 signaling pathway, (GO: 0018108) peptidyl-tyrosine phosphorylation, (GO: 0043406) positive regulation of MAP kinase activity, (GO: 0043627) response to estrogen, (GO: 0071364) cellular response to epidermal growth factor stimulus, (GO: 0060397) JAK-STAT cascade involved in growth hormone signaling pathway, (GO: 0043536) positive regulation of blood vessel endothelial cell migration, (GO: 0051092) positive regulation of NF-kappaB transcription factor activity, (GO: 0007265) Ras protein signal transduction, (GO: 0048010) vascular endothelial growth factor receptor signaling pathway, (GO: 0050729) positive regulation of inflammatory response, (GO: 0042517) positive regulation of tyrosine phosphorylation of Stat3 protein, (GO: 0043401) steroid hormone mediated signaling pathway, (GO: 0042523) positive regulation of tyrosine phosphorylation of Stat5 protein, (GO: 0060070) canonical Wnt signaling pathway, (GO: 0007179) transforming growth factor beta receptor signaling pathway, (GO: 0007169) transmembrane receptor protein tyrosine kinase signaling pathway, (GO: 0014065) phosphatidylinositol 3-kinase signaling, (GO: 0033209) tumor necrosis factor-mediated signaling pathway, (GO: 0032570) response to progesterone, (GO: 0000186) activation of MAPKK activity, (GO: 0043123) positive regulation of I-kappaB kinase/NF-kappaB signaling, (GO: 0007173) epidermal growth factor receptor signaling pathway, (GO: 0033598) mammary gland epithelial cell proliferation, (GO: 0048009) insulin-like growth factor receptor signaling pathway, (GO: 0030330) DNA damage response, and signal transduction by p53 class mediator.

Cluster 6 has (GO: 0008202) steroid metabolic process, (GO: 0007223) Wnt signaling pathway, calcium modulating pathway, (GO: 0008283) cell proliferation, and (GO: 0043401) steroid hormone mediated signaling pathway.

Analyzing another cluster by the same way, we get the same related biological processes. The details are described in Table S8.

Meanwhile, we get eighteen ER-positive breast cancer-related pathways. They are the same as pathways in Figures 3 and 7, which prove that these pathways may be the key pathways in ER-positive breast cancer's development. The details are described in Table S9.

**Figure 11:**
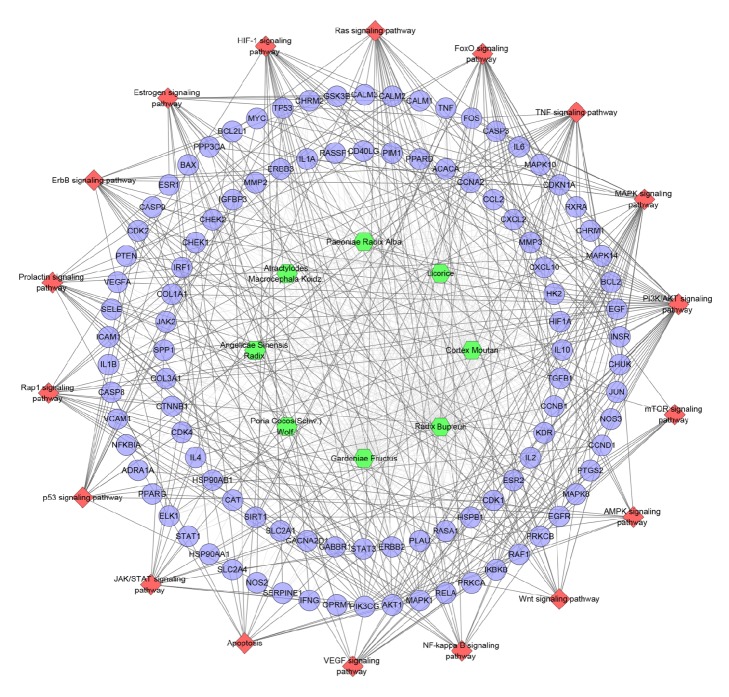
Pathway of compound-known target-ER-positive breast cancer network (blue circle stands for compound target; red diamond stands for pathway; green hexagon stands for herb).

Overall, although the potential anticancer compounds have been shown to have a therapeutic effect on breast cancer, respectively, through this network analysis, this still needs further research. Through this network relationship, we can select the real anticancer compounds in DXP and explore the pharmacological and molecular mechanism of DXP treating ER-positive breast cancer. Through our research, we can find that Ras signaling pathway, MAPK signaling pathway, PI3K/AKT signaling pathway, ErbB signaling pathway, Prolactin signaling pathway, Hippo signaling pathway, HIF-1 signaling pathway, FoxO signaling pathway, and so on may be the focus of the future study of the effect of DXP formula and its compounds (Figures 3, 7, and 11). Meanwhile, it is reported that glabridin combined tamoxifen has potential to be used as an estrogen replacement drug and may reduce the risk of endometrial cancer that has arisen from the intake of tamoxifen [83], and a research shows that quercetin may reverse tamoxifen resistance in tamoxifen-resistant breast cancer cells by the upregulation of ER*α* combined with downregulation of HER-2 [84]. This suggests that DXP or some of its compounds may be used with chemotherapy drugs to reduce drug resistance and increase efficacy, so as to reduce the use and toxicity of chemotherapy drugs. This also gives us a new idea that when these compounds (or drugs) are used in combination, the therapeutic effect and targeting effect are better.

## 4. Conclusions

Currently, SERMs, SERDs, and so on are still the pharmacology options for breast cancer in clinical practice. Our research finds that some of the compounds in DXP may play an anticancer role, such as pachymic acid quercetin, ergosterol peroxide, and licochalcone B. In this study, a number of network-based computational methods and algorithm-based approaches to predict targets, collect known targets, and construct networks are combined to predict, illuminate, and confirm the molecular synergy of DXP for ER-positive breast cancer. This method provides clues to the researcher who explores ethnopharmacological or/and herbal medicine's or even multidrugs' various synergies. We also successfully found the potential ER-positive breast cancer associated targets, cluster, biological processes, and pathways. The ER-positive breast cancer network and DXP-ER-positive breast cancer network had shown the probable molecular mechanism of ER-positive breast cancer's development and the potential pharmacological and molecular mechanism of DXP treating this breast cancer. And the compound-known target-ER-positive breast cancer network confirmed these mechanisms and indirectly proved the rationality of herb combinations of DXP.

## Figures and Tables

**Figure 8:**
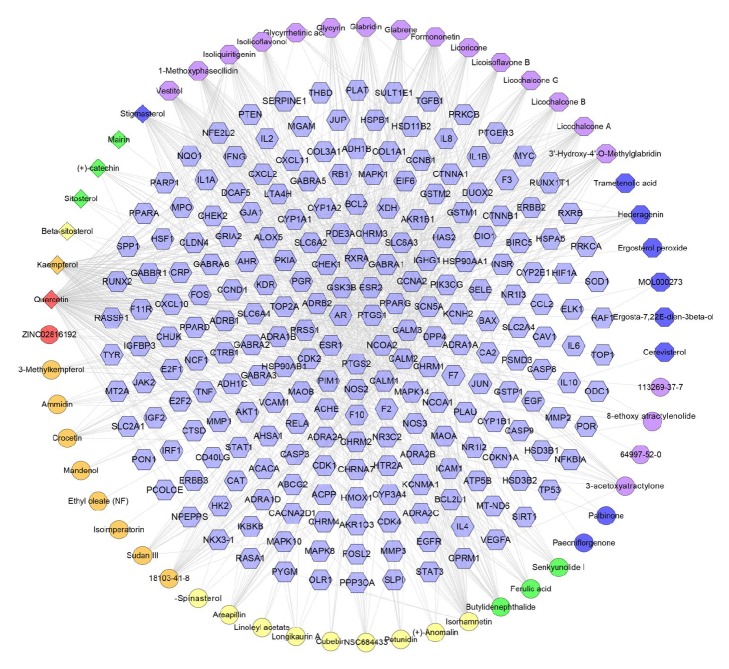
Compound-known target network (blue hexagon stands for known target; the representation of red, orange, yellow, green, blue, and purple circle and blue, green, yellow, orange and red diamonds is the same as Figure 4; blue and purple octagon stand for compounds of* Poria Cocos (Schw.) Wolf.* and* Licorice*).

**Table 1:** Cluster of ER-positive breast cancer PPI network.

Cluster	Score	Nodes	Edges	Genes
1	17	19	153	EIF4E, MTOR, EGFR, ERBB3, PIK3CA, SRC, PGR, IGF1, ESR1, CCND1, ERBB2, TP53, BCL2, FOXO3, JAK2, STAT5B, MYC, KDR, PTGS2
2	3	7	9	FOXA1, RUNX1, NCOR2, GATA3, TFF1, EZH2, SIRT1

**Table 2:** Cluster of herbal formula-disease PPI network.

Cluster	Score	Nodes	Edges	Genes
1	30.486	38	564	CCND1, STAT1, IGF1, MAP2K1, PIK3CA, BCL2, F2, PTK2, MYC, JAK2, RHOA, PIK3R1, BCL2L1, AKT2, NOS3, AKT1, IGF1R, HMOX1, MMP9, ABL1, KIT, RAF1, IL2, CDC42, GRB2, LCK, MMP2, KDR, ERBB2, ERBB3, STAT5B, RAC1, TP53, PTPN11, MAPK8, MTOR, FOXO3, MET

2	16	52	408	Ergosterol peroxide, ethyl oleate (NF), PPARD, PAK1, PDPK1, EPHA2, EGF, RET, SYK, RARA, CCL2, WT1, YAP1, NOS2, glabridin, glycyrrhetinic acid, NME2, BRAF, ZAP70, PPARG, ESRRG, PPARA, HSP90AA1, HCK, CASP3, linoleyl acetate, longikaurin A, mairin, mandenol, CDK6, MOL000273, MOL001910, 508-02-1, 64997-52-0, 8*β*-ethoxy atractylenolide III, pachymic acid, paeonidanin, palbinone, ESR2, CSK, saikosaponin C, MDM2, PGR, beta-sitosterol, supraene, trametenolic acid, troxerutin, RORA, *α*-amyrin, FGF1, EGFR, NCOR2

3	10.421	20	99	XIAP, ESR1, MAPK1, FGFR1, MAPK14, MMP1, RAC2, MYB, PTGS2, PTPN1, MAPK12, EIF4E, AR, SRC, ERBB4, INSR, JAK3, GSK3B, SIRT1, ALB

4	9.143	43	192	NR1H2, F7, ferulic acid, MAPK10, BTK, MOL000285, RARG, 4-O-methylpaeoniflorin, APAF1, THRA, CASP1, GATA3, MUC1, PLK1, HSP90AB1, PRKACA, PLG, VDR, CTSD, RUNX1, glabrene, CHEK1, PRKCQ, LGALS3, poricoic acid A, glycyrrhizin, ITK, FGFR2, EZH2, RARB, HSPA8, TNFRSF11B, CASP7, sudan III, CA2, ZINC02816192, THRB, HRAS, HNF4G, kaempferol, TEK, licochalcone G, NCOA3

5	7.829	36	137	APRT, IMPDH2, MMP3, NR3C1, NR1H3, ELANE, 7,9(11)-dehydropachymic acid, GMPR2, paeoniflorgenone, paeoniflorin sulfonate, PGF, areapillin, UMPS, quercetin, senkyunolide I, stigmasterol, ARF1, CALM2, DHFR, TGFB2, butylidenephthalide, isoliquiritigenin, (+)-anomalin, isorhamnetin, vestitol, crocetin, 113269-36-6, CANT1, *α*-spinasterol, ANXA5, NR1I2, ARHGAP1, licochalcone A, 113269-37-7, licoricone, MTHFD1

6	6.368	39	121	MMP8, PARP1, GSTM1, RAN, PPP5C, 3*β*-acetoxyatractylone, PPIA, CYP2C8, CCNA2, HPRT1, GSS, SULT2A1, BIRC7, 113269-39-9, SETD7, EPHB4, DHODH, REN, GSTO1, petunidin, DPP4, DUT, OTC, GSTT2, ADK, DTYMK, CALM1, PAPSS1, TK1, IMPDH1, hederagenin, MKI67, CDK7, GSTA1, GSTP1, CTSB, AURKA, licochalcone B, MAPKAPK2

7	4.848	34	80	Dehydroeburicoic acid, ergosta-7,22E-dien-3beta-ol, RAP2A, MOL000280, MOL000287, mudanpioside H, HDAC4, NSC684433, octalupine, 18103-41-8, RXRA, PLAU, formononetin, albiflorin, 1-methoxyphaseollidin, HSPD1, paeoniflorin, KAT2B, TOP2A, glycyrin, ammidin, poricoic acid B, poricoic acid C, CTNNA1, sainfuran, sitosterol, isoimperatorin, isolicoflavonol, cerevisterol, 3-methylkempferol, RND3, CDK2, licoisoflavone B, cubebin

8	4.762	22	50	SDS, SHMT1, CTSG, AK1, ACE, TYMS, GMPR, MME, GP1BA, METAP2, DCK, PIP, (+)-catechin, ATIC, GART, FKBP1A, PSPH, CBS, ITGAL, NT5M, LYZ, PDE4D

9	3.538	14	23	G6PD, RAB5A, ARF4, LDHB, BACE1, AHCY, PKLR, PYGL, RAB11A, HK1, ADAM17, GSR, MMP13, SOD2

10	3.333	4	5	GALK1, GPI, GCK, ME2

11	3	3	3	TPH1, CBR1, SPR

**Table 3:** Cluster of known target-breast cancer PPI network.

Cluster	Score	Nodes	Edges	Genes
1	44.125	49	1059	MAPK8, MMP1, STAT1, HMOX1, F2, AR, PPARG, ICAM1, NOS3, KDR, SRC, MTOR, STAT5B, MAPK14, FOXO3, IGF1, STAT3, CCND1, MAPK1, PGR, EGFR, VEGFA, BCL2L1, CDKN1A, MMP2, IL6, TP53, RAF1, HIF1A, ERBB2, MYC, JUN, IL8, BIRC5, TGFB1, IL2, SERPINE1, CTNNB1, PTEN, FOS, PPARA, JAK2, RELA, ERBB3, ESR1, HSP90AA1, AKT1, BCL2, TNF
2	10	10	45	CXCL11, ADRA2A, CHRM2, CXCL2, ADRA2C, OPRM1, CHRM4, ADRA2B, CXCL10, PTGER3
3	8	22	84	ERBB4, CAT, VCAM1, CDK1, BAX, IL1B, CCL2, Quercetin, IL4, SIRT1, RUNX2, GSK3B, HSPB1, INSR, CDK2, PIK3CA, PTGS2, IGFBP3, IL10, CCNB1, RB1, CDK4
4	3.9	21	39	CASP3, HSPA5, WT1, RUNX1, CHEK2, CAV1, AHR, FOSL1, ESR2, MMP3, NCOA3, SLC2A4, PIK3CG, EIF4E, IRF1, MYB, NFKBIA, IKBKB, CHEK1, GJA1, CASP8
5	3.882	18	33	Hederagenin, CALM3, GABRA2, GABRA5, GABRA6, stigmasterol, GABRA3, ADH1B, 1-methoxyphaseollidin, licoricone, CYP1A2, GABRA1, isorhamnetin, KCNH2, GSTM2, XDH, 18103-41-8, KCNMA1
6	3.429	15	24	RXRA, CHRM3, ADRA1A, CYP3A4, NR1I2, ADRA1B, ADRA1D, GSTM1, F10, glabridin, PLAT, HTR2A, CYP2E1, CALM1, CALM2
7	3.333	4	5	CYP1A1, NFE2L2, kaempferol, GSTP1
8	3	15	21	ABCG2, NOS2, MAPK10, HSF1, PLK1, CHUK, TOP2A, CCNA2, NCOR2, IFNG, GATA3, IL1A, HDAC4, TOP1, EZH2
9	3	3	3	PLAU, E2F1, E2F2
10	3	3	3	PPP3CA, PIM1, ACACA
11	3	3	3	SLC6A2, ferulic acid, MAOB

## Abbreviations

ER: Estrogen receptor
PGR: Progesterone receptor
HER: Human epidermal growth factor receptor
SERM: Selective estrogen receptor modulator
SERD: Selective estrogen receptor downregulator
AI: Aromatase inhibitors
CAM: Complementary and alternative medicine
TCM: Traditional Chinese medicine
DXP: Danzhi Xiaoyao powder
OB: Oral bioavailability
DL: Drug-likeness
PPI: Protein-protein interaction
GO: Gene Ontology
ERE: Estrogen responsive element
IGF: Insulin-like growth factor
IGF1R: IGF-1 receptor
MAPK: Mitogen-activated protein kinase
STAT: Signal transducers and activators of transcription
EGF: Epidermal growth factor
EGFR: EGF receptor
HB-EGF: Heparin-binding EGF-like growth factor
IRS: Insulin receptor substrate
TGF: Transforming growth factor
PI3K: Phosphoinositide 3-kinase
GPR30: G-protein coupled estrogen receptor
GFR: Growth factor receptor
RTK: Receptor tyrosine kinase.

## Supplementary Materials

Supplementary Materials Table S1: compound targets for DXP. Table S2: known targets for DXP. Table S3: ER-positive breast cancer targets. Table S4: enrichment analysis of clusters based on Gene Ontology (GO) annotation. Table S5: pathway enrichment analysis. Table S6: enrichment analysis of clusters based on Gene Ontology (GO) annotation. Table S7: pathway enrichment analysis. Table S8: enrichment analysis of clusters based on Gene Ontology (GO) annotation. Table S9: pathway enrichment analysis.
